# Supplementary material for: Myoscaffolds reveal laminin scarring is detrimental for stem cell function while sarcospan induces compensatory fibrosis
Source: NPJ Regen Med. 2023 Mar 15;8:16. doi: 10.1038/s41536-023-00287-2 (PMC10017766; doi:10.1038/s41536-023-00287-2)
Supplement: Supplementary file 1 — Supplementary Information [file 41536_2023_287_MOESM1_ESM.pdf]

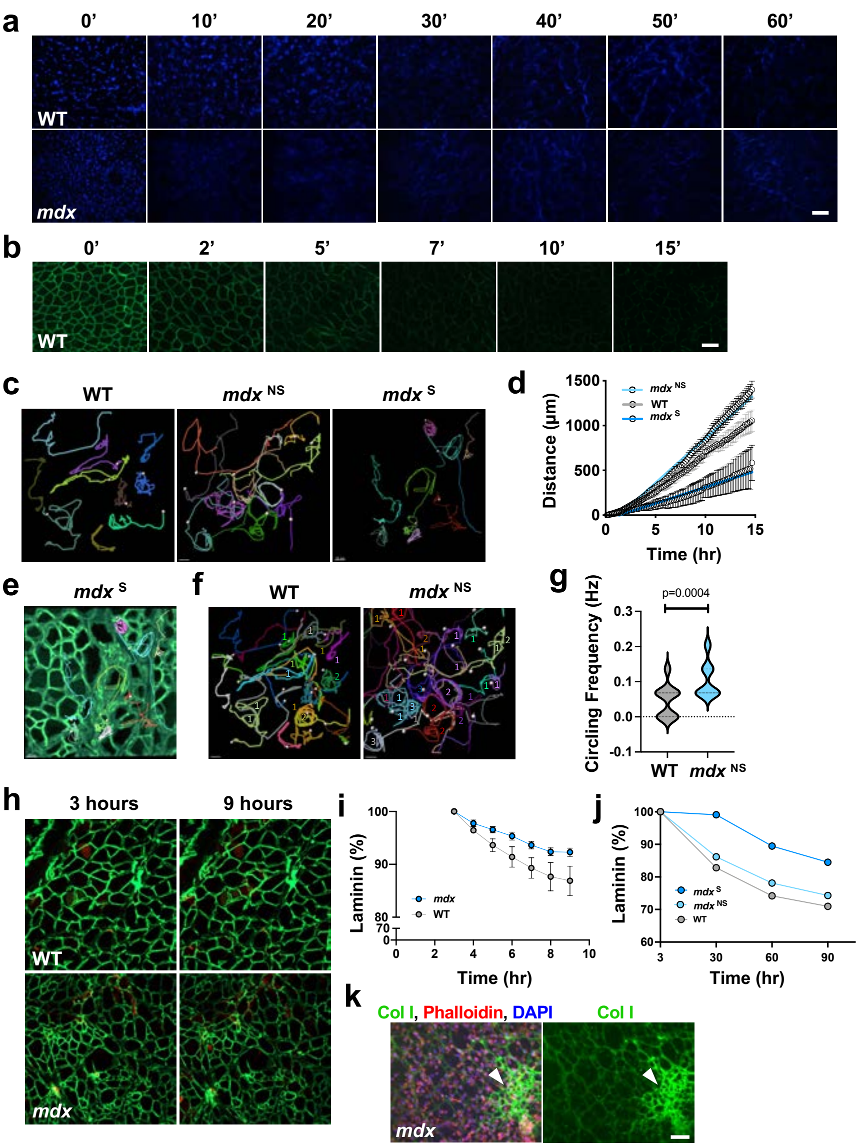

Supplementary Figure 1. Myoscaffolds from healthy and diseased muscle differentially support SMPC migration and growth.
a. Representative images of DAPI staining (blue) performed on transverse cryosections from wild-type (WT) and *mdx* quadriceps muscles that were decellularized with 1% SDS solution for the indicated times (10’ to 60’). Non-decellularized (whole) muscle sections were used as controls and are denoted as the 0’ time point. Scale bar, 100 µm. (n=3 independent experiments) **b.** Immunofluorescence analysis of dystrophin (green) shows rapid removal of the protein within 15 minutes of 1% SDS treatment (indicated times 2’ to 15’). Non-decellularized (whole) muscle sections were used as controls (0’) (n=3 independent experiments). Scale bar, 100 µm. **c.** Individual SMPC tracks of total displacement are marked by unique colors and highlight SMPC behavior differences on *mdx* myoscaffolds (WTC-11 cells). **d.** Total cell displacement (microns) was calculated by combining cell speed with cumulative sum of speeds from previous time points. Non-constrained linear regression was used to calculate the slope of displacement (WTC-11 cells, n=10-14 cells/tissue; based on observations from n=3 independent experiments) **e.** Overlay of the cell tracks from panel (**c**) with the image of the *mdx*^{S} region from live cell imaging (Video S3) showing limited cell motility over fibrotic scars. **f.** The frequency at which SMPCs circled the basement membrane was calculated for individual SMPCs on WT and *mdx*^{NS} myoscaffolds (WTC-11 cells, n=19-22 cells/tissue; based on observations from n=3 independent experiments). The white dots represent the final location of the cell following the 15-hour tracking period. **g.** Circling frequency was calculated as the number of times a cell circled the basement membrane over a 14 hour period. Differences in frequency were calculated using an independent samples t-test (p<0.05). We found that cells on *mdx*^{NS} myoscaffolds circled the basement membrane at a 2.3 fold greater frequency than those on WT myoscaffolds (WT: 0.047 ± 0.046 vs. *mdx*^{NS}: 0.105 ± 0.050 Hz (mean ± st. dev.), p=0.0004) (WTC-11 cells, n=19-22 cells/tissue). **h.** Representative immunofluorescent images of WT and *mdx* myoscaffolds cultured with SMPCs (WTC-11 hiPSCs) taken at 3 and 9 hours after cell seeding (laminin alpha 2-green, SMPCs- red). **i.** Mean laminin intensity was calculated each hour over the entire field of view, starting after 3 hours, which was the time required for the image to stabilize as cells settled and migrated on the myoscaffolds. Although not significant, there was a trend toward a greater decrease in laminin intensity in WT myoscaffolds, compared to *mdx*, indicating greater laminin degradation (n=4 samples/group, mean +/- SEM). **j.** For a single experiment, laminin fluorescence intensity was quantified over 90 hours of imaging, starting 3 hours after cell seeding. Laminin intensity began to drop immediately on WT and *mdx*^{NS} myoscaffolds, suggestive of laminin degradation, while laminin intensity dropped only 1% in the first 30 hours when cells were cultured on *mdx*^{S} regions (n=1 region/tissue type, based on observations from n=3 independent experiments). **i.** Representative image of an *mdx* myoscaffold with a fibrotic scar (white arrowhead), stained for collagen I (Col I, green), phalloidin (red), and DAPI (blue). Collagen I in the fibrotic scar was resistant to remodeling by SMPCs, as indicated by increased fluorescence in the fibrotic region relative to the rest of the myoscaffolds (n=4 independent experiments). Scale bar, 100 µm.

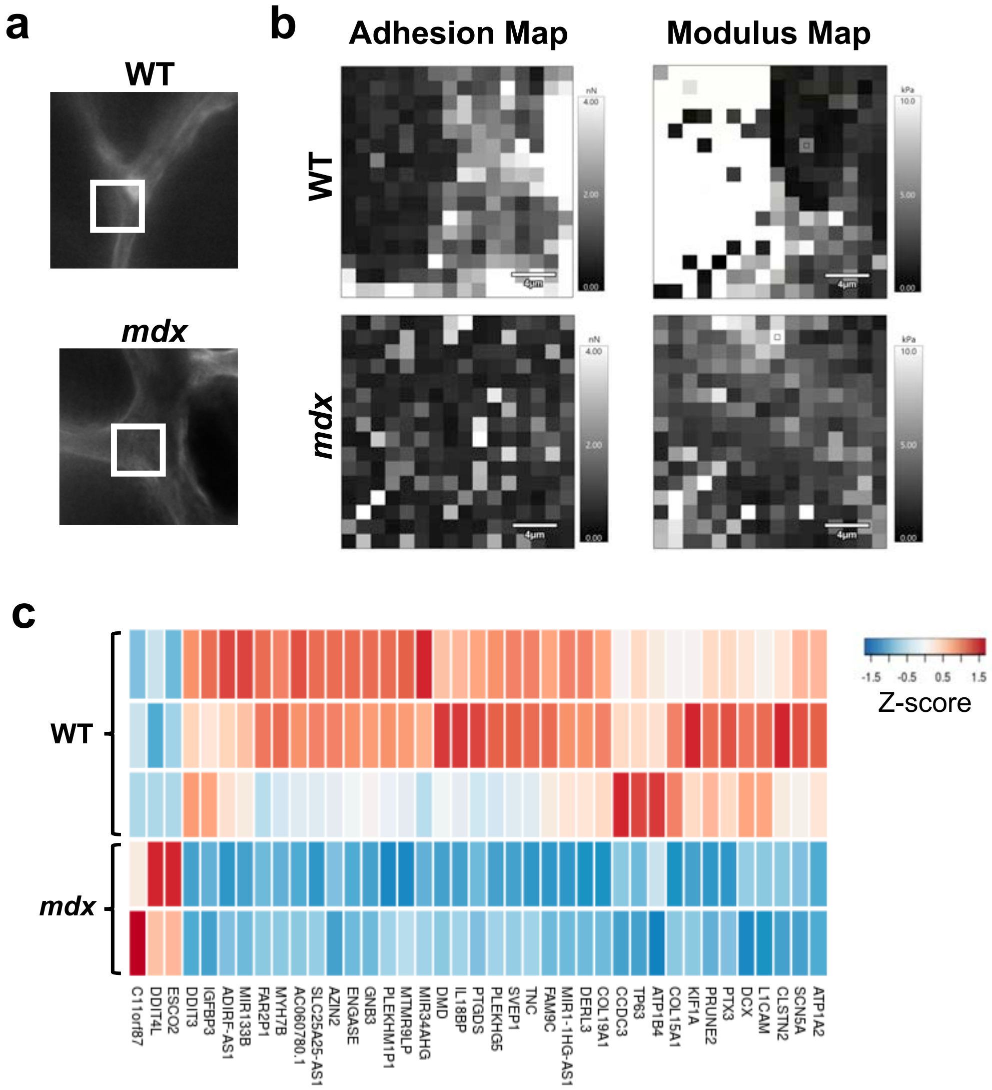

Supplementary Figure 2. Stiff *mdx* myoscaffolds have reduced adhesive properties and induce downregulation of genes associated with cell adhesion, migration, and skeletal muscle maturation.

a. Images of the WT and *mdx* ECM samples from AFM testing stained for laminin using indirect immunofluorescence. The white box represents the 20 x 20μm region scanned from each sample. **b.** Representative adhesion and modulus maps from the samples shown in (**a**) (n=3 independent experiments). **c.** RNA sequencing was performed using total RNA prepared from SMPCs cultured (5 days) on WT and *mdx* myoscaffolds. Expression patterns of differentially expressed genes (DEGs) from SMPCs (H9 cells) cultured on WT (n=3) and *mdx* (n=2) myoscaffolds. Expression levels are shown as Z-scores, as in scale. Most DEGs were downregulated in *mdx* compared to WT, including L1 cell adhesion molecule (L1CAM) and calsyntenin (CLSTN2) that play roles in cell adhesion and migration. Genes associated with skeletal muscle development and maturation such as myosin heavy chain 7B (*MYH7B*), dystrophin (*DMD*), tenacin C (*TNC*), and kinesin family member 1A (*KIF1A*) were also downregulated. Several cell adhesion genes including integrins (*ITGA4, ITGA6*), M-cadherin (*CDH15*), and neural cell adhesion marker (*NCAM1*) as well as sarcomeric genes (myosin heavy chain 3 and 7 (*MYH3, MYH7*) and troponin T2 (*TNNT2*) trended toward downregulation in SMPCs cultured on *mdx* myoscaffolds ($p<0.05$). Genes associated with basement membrane assembly, including collagens type XV and XIX (COL15A1 and COL19A1), exhibited decreased expression in *mdx* samples.

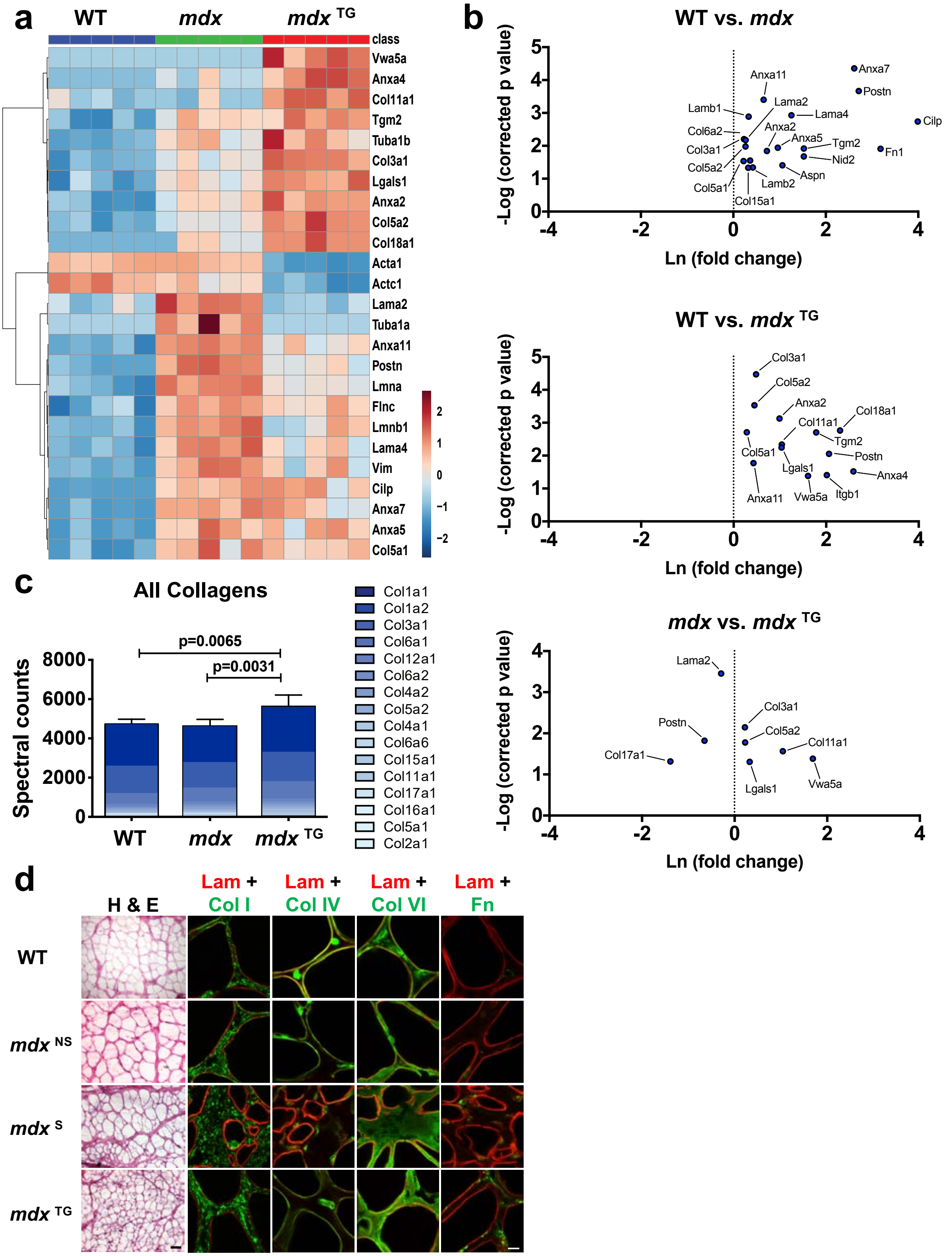

Supplementary Figure 3. The matrisome of *mdx* and *mdx* ^{TG} samples diverge from that of wild-type muscle.
a. Heat map from ECM focused proteomics reveals distinct clustering of each phenotype (n=5 samples/group). **b.** Volcano plots showing ln fold change plotted against −log10 adjusted P value for WT vs. *mdx,* WT vs. *mdx* ^{TG}, and *mdx* vs. *mdx* ^{TG} samples. **c.** Column graphs showing the abundance of collagens in skeletal muscle from WT, *mdx*, and *mdx* ^{TG} samples. *P* values reflect analysis by one-way ANOVA. **d.** Replicate images from analysis of the abundance and organization of ECM components in the basement membrane and interstitial matrix regions of WT, *mdx*, and *mdx* ^{TG} samples. Regions of *mdx* myoscaffolds without fibrotic scars (*mdx* ^{NS}) and with scars (*mdx* ^{S}) shown separately. Representative images are shown from H&E staining, along with indirect fluorescent image analysis of myoscaffolds co-stained for laminin α2 (Lam) (red) with collagen I (Col I), IV (Col IV), VI (Col VI), and fibronectin (Fn) (green), respectively (selected images from n=4 independent experiments). Scale bars, 100µm (H&E) and 8µm (IFA).

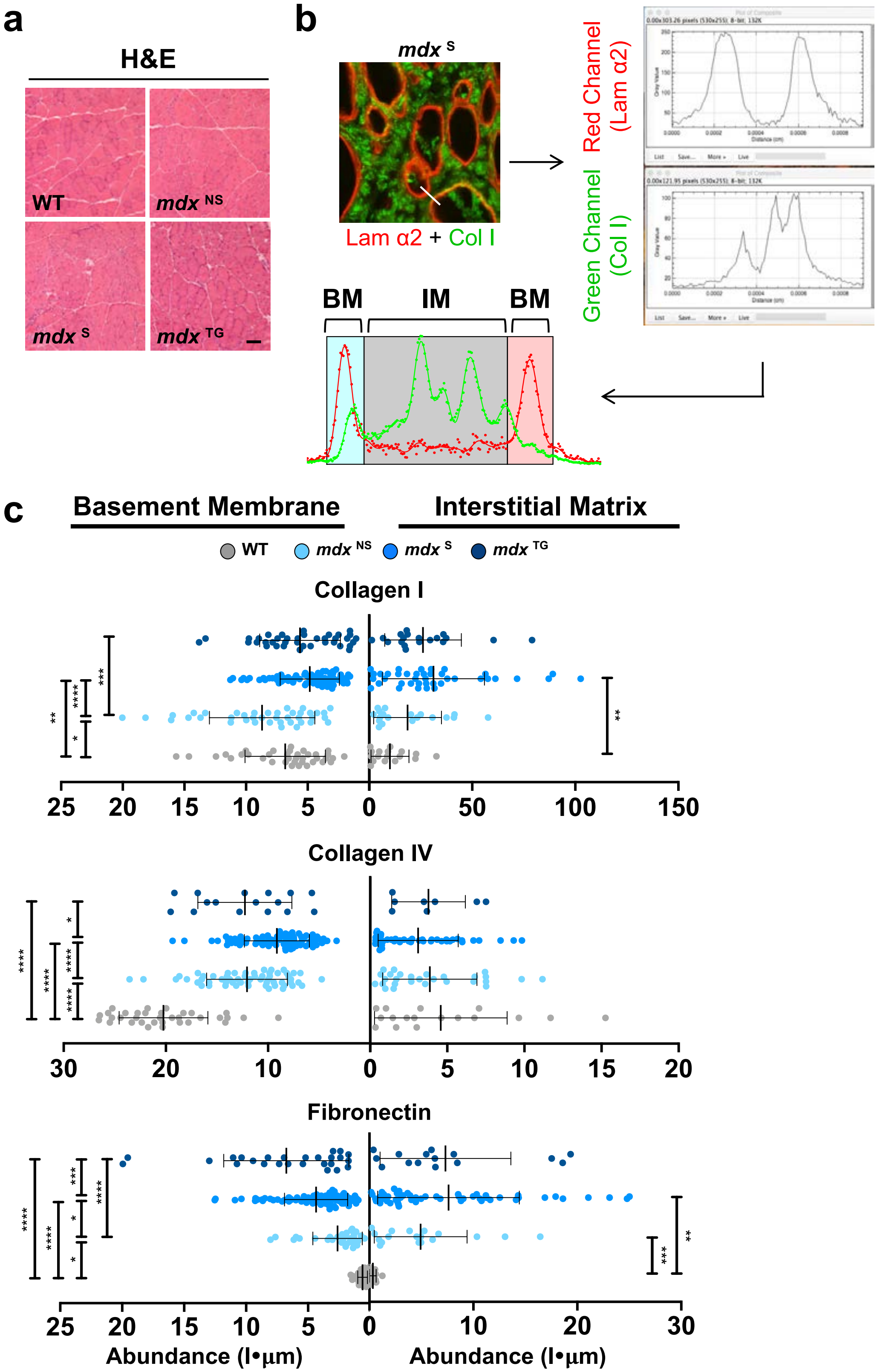

Supplementary Figure 4. Quantification of ECM protein abundance and distribution reveals alterations in healthy and diseased tissues. **a.** Representative images of transverse sections of the quadriceps muscle from WT, *mdx* and *mdx* ^{TG} mice stained with H&E to visualize muscle pathology. **b.** To quantify the distribution of ECM proteins in the basement membrane (BM) and interstitial matrix (IM) of co-stained myoscaffolds, a line was drawn across the endomysium (white bar) on confocal images and the line scan function in Image J was used to generate plot profiles of the pixel intensity for each ECM protein (sample confocal image of an *mdx* ^{S} myoscaffold: red channel-laminin α2, green channel-collagen I). The plot profiles were exported into Matlab and a custom algorithm was utilized to calculate the abundance of each protein in the BM and IM regions. **c.** Graphs showing the abundance of collagens I and IV and fibronectin in the BM and IM of WT, *mdx* ^{NS}, *mdx* ^{S}, and *mdx* ^{TG} ECM (n=20-40 measurements/group for each protein, n=4 independent experiments). Between group differences were analyzed by one-way ANOVA. *P* values are as follows: * = *p<0.05*, ** = *p<0.01*, *** = *p<0.001*, **** = *p<0.0001*.

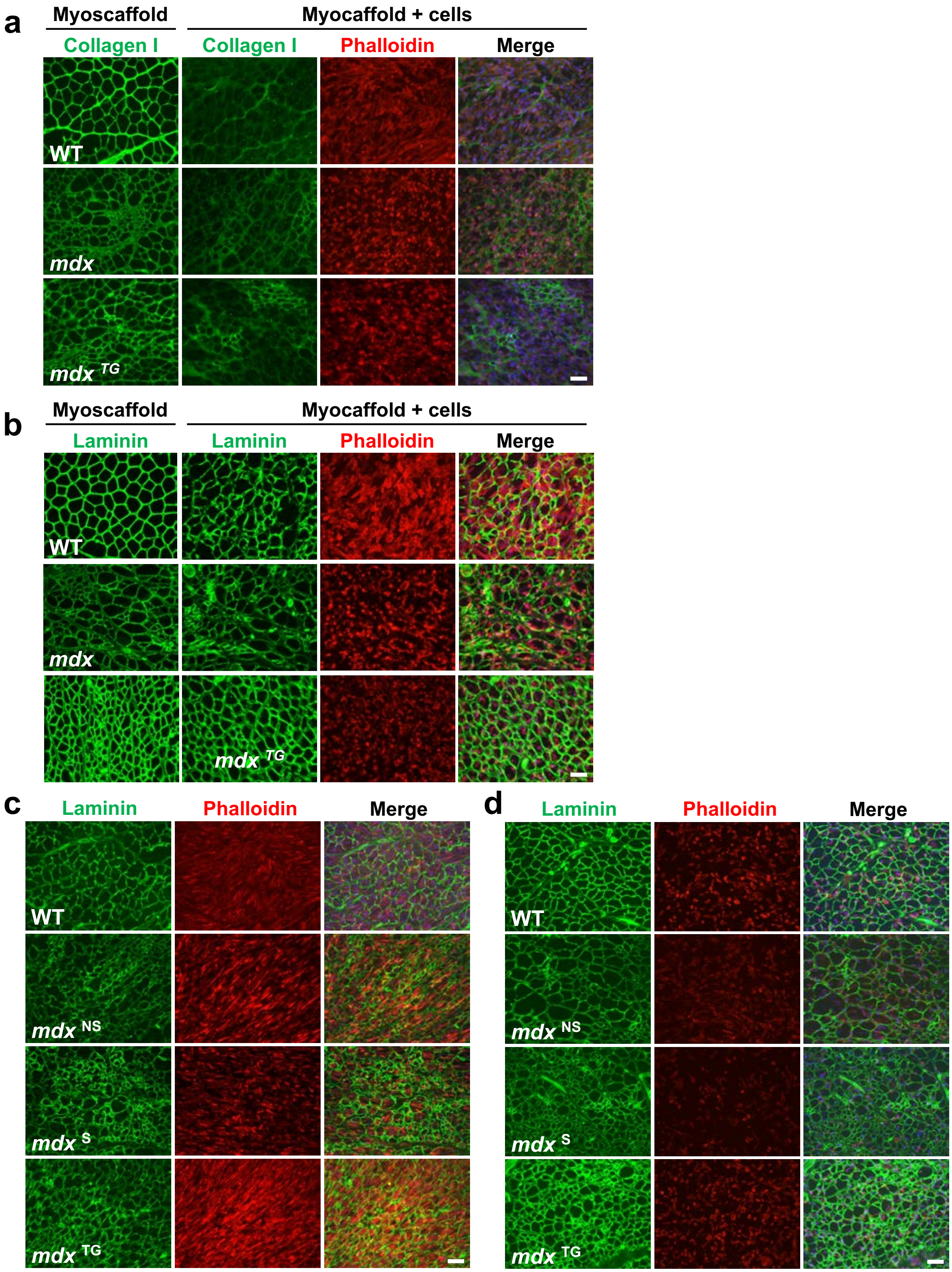

Supplementary Figure 5. SMPCs do not remodel laminin in *mdx* fibrotic scars.
a-b. Indirect immunofluorescence confocal microscopy of SMPCs (CDMD 1002) cultured for 5 days on WT, *mdx,* and *mdx* ^{TG} myoscaffolds (myoscaffold + cells) stained with antibodies recognizing collagen I (**a**) or laminin (**b-d**) (green), along with phalloidin (red) and DAPI (blue). The fluorescence intensity was only controlled between the myoscaffolds and myoscaffolds + cells condition for each group (WT, *mdx,* and *mdx* ^{TG}), therefore laminin and collagen I abundance cannot be compared between groups. Scale bar, 100µm. **c-d.** Immunofluorescent images from replicate experiments performed with SMPCs (H9) and stained as above. The same muscle samples were used to generate the myoscaffolds shown in panels (**c**) and (**d**), but 2 different clones of the H9 cell line were used (clone 1 in panel **c**, clone 2 in panel **d**). Although the two clones exhibit variable cell proliferation and laminin remodeling capacity, they demonstrate similar trends in laminin remodeling and cell morphology, including reduced laminin remodeling on *mdx* ^{S} myoscaffolds.

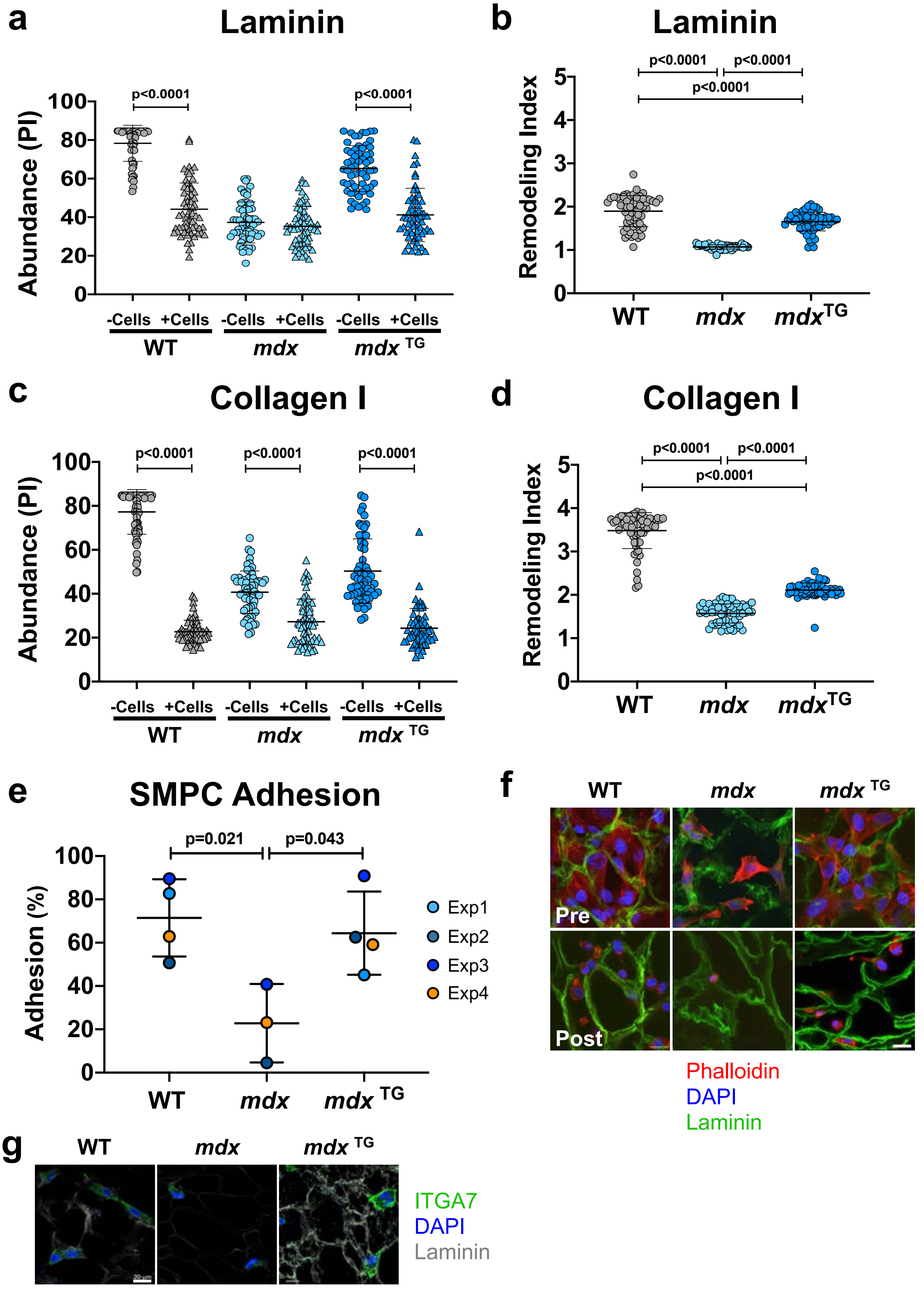

Supplementary Figure 6. Improved SMPC adhesion to *mdx* ^{TG} myoscaffolds accompanies improved laminin remodeling and is facilitated by increased expression of Itga7.

a-d. WT, *mdx*, and *mdx* ^{TG} myoscaffolds without (- cells) and with SMPCs (+ cells, CDMD 1002 cells) were cultured in proliferation media (5 days) and then stained with antibodies against laminin and collagen I. Protein abundance (maximum pixel intensity (PI)) was measured from 60 endomysial locations/ myoscaffold. The remodeling index (RI) was calculated as the ratio of protein abundance (PI) in the myoscaffolds in the absence of cells to protein abundance in the myoscaffolds after cell seeding. SMPCs were unable to remodel laminin in *mdx* scaffolds (**a**), with an RI value close to 1 (**b**). While all SMPCs remodeled collagen I (**c**), the RI was significantly lower for cells on *mdx* myoscaffolds (**d**). **e.** Average SMPC adhesion to WT, *mdx*, and *mdx* ^{TG} myoscaffolds was calculated from n=4 independent experiments using both CDMD 1002 (Exp1) and H9 cell lines (Exp2, Exp3, and Exp4). A significant reduction in cell adhesion was observed with culture on *mdx* scaffolds, while there was no difference observed between WT and *mdx* ^{TG} across all cell lines. **f.** Images of SMPCs (H9 cells) cultured for 4 hours on WT, *mdx*, and *mdx* ^{TG} myoscaffolds both before (pre) and after (post) exposure to dissociation buffer. Scale bar, 20 µm. **g.** An increased abundance of integrin α7 (Itga7) was observed in SMPCs (H9 cells) cultured for 4 hours on *mdx* ^{TG} myoscaffolds, compared to those on *mdx* scaffolds. Scale bar, 20 µm. (n=3 independent experiments)

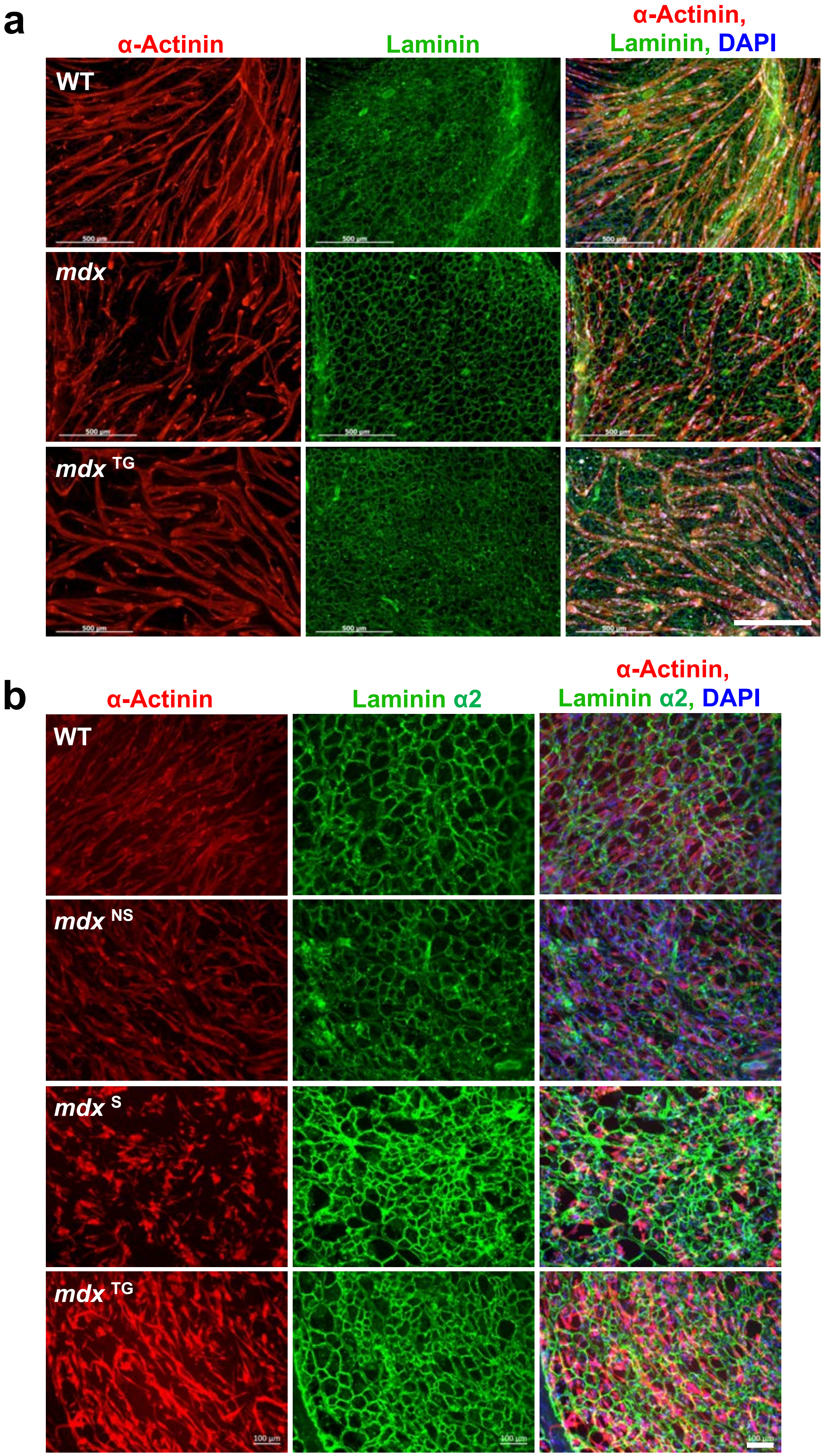

Supplementary Figure 7. SMPC differentiation is inhibited on *mdx* myoscaffolds.
a-b. Replicate SMPC differentiation experiments performed using CDMD 1002 (**a**) and H9 (**b**) cell lines. Cell differentiation is inhibited on *mdx* ^{S} myoscaffolds, while both cell lines demonstrate robust myotube formation on *mdx* ^{TG} myoscaffolds. Scale bar, 500µm (**a**) and 100µm (**b**).

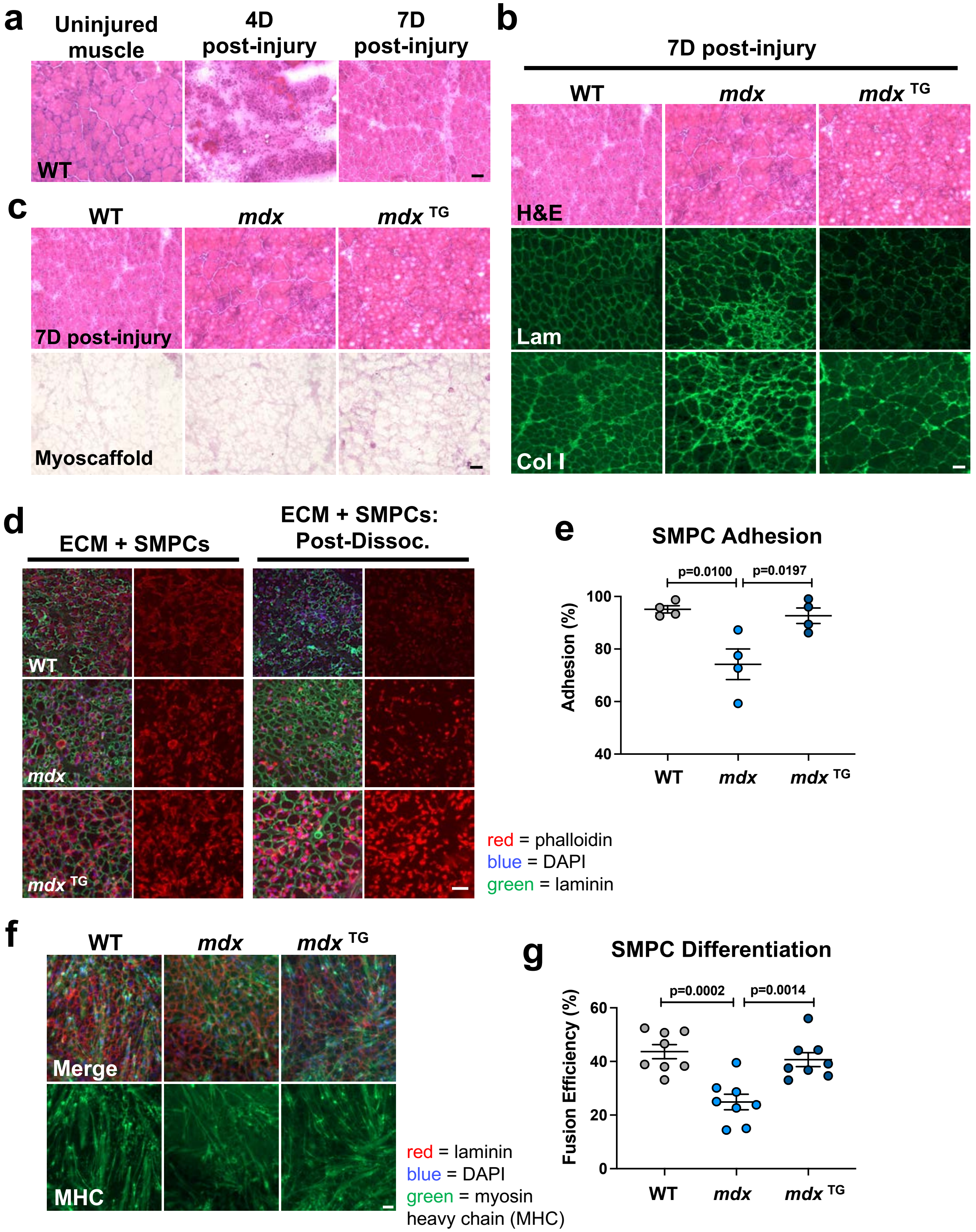

Supplementary Figure 8. Reduced SMPC adhesion and fusion efficiency on *mdx* myoscaffolds derived from $BaCl_2$ treated muscle. **a.** Images of H&E stained transverse muscle sections from the tibialis anterior (TA) of wild-type (WT) mice; uninjured and following $BaCl_2$ injection (4 and 7 days post-injury). Scale bar=50µm. **b.** H&E and immunofluorescent images of TA muscle sections from WT, *mdx*, and *mdx* ^{TG}, taken at 7 days post-injury. Scale bar=50µm. **c.** H&E images showing whole muscle (7D post-injury) and a muscle section decellularized in 1% SDS for 10 minutes (Decell), from WT, *mdx*, and *mdx* ^{TG} TA muscles taken at 7 days post-injury. Cellular material is effectively removed after 10 minutes while ECM architecture is maintained. Scale bar=50µm. **d.** Images of SMPCs (H9 cells) cultured for 4 hours on WT, *mdx*, and *mdx* ^{TG} myoscaffolds both without (ECM + SMPCs) and with (ECM + SMPCs: Post-Dissoc) exposure to dissociation buffer (n=4/group, H9 SMPCs). **e.** A significant reduction in cell adhesion was observed on *mdx* myoscaffolds relative to both WT and *mdx* ^{TG} (n=4/group). Scale bar=100µm. **f.** Immunofluorescent images of SMPCs cultured in proliferation media and then differentiated on WT, *mdx*, and *mdx* ^{TG} myoscaffolds (n=4/group, H9 SMPCs). Scale bar=50µm. **g.** Fusion efficiency was significantly reduced on *mdx* myoscaffolds (fusion efficiency calculated from n=4/group, 2 locations per tissue).

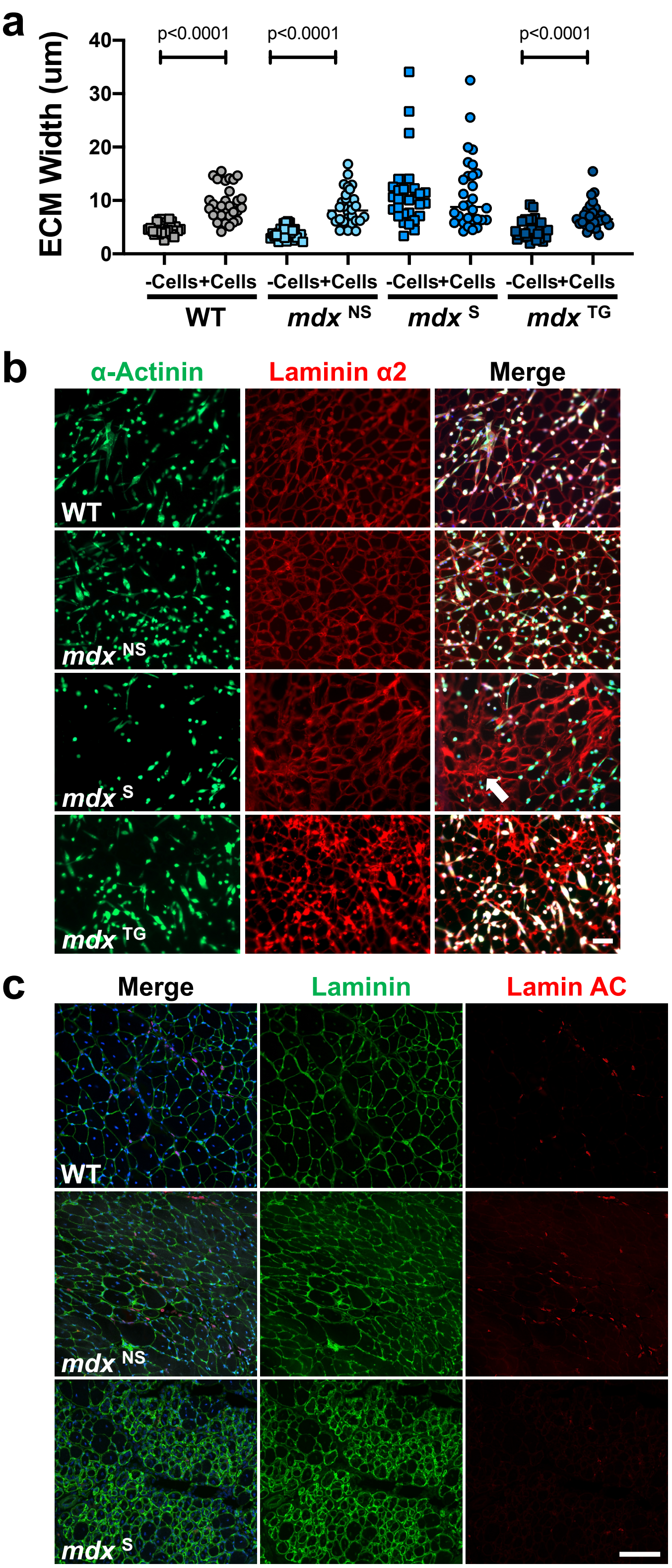

Supplementary Figure 9. Quantification of endomysial width reveals limited satellite-cell mediated ECM remodeling of *mdx* ^{S} myoscaffolds while limited SMPC engraftment is observed in *mdx* ^{S} *in vivo*.
a. Confocal images of murine satellite cells differentiated on WT, *mdx* ^{NS}, *mdx* ^{S}, and *mdx* ^{TG} myoscaffolds (+Cells), as well as control myoscaffolds without cells (-Cells) were utilized to measure endomysial width from 20 locations/image (line drawing tool, Image J) (n=3 samples/group). Myoscaffolds were labeled with antibodies against laminin to demarcate the basement membrane. There was a significant increase in the width of the endomysium in all tissues following cell differentiation, except the *mdx* ^{S} myoscaffolds. **b.** Confocal images from replicate cell differentiation experiments utilizing ZsGreen fluorescent murine satellite cells cultured on WT, *mdx* ^{NS}, *mdx* ^{S}, and *mdx* ^{TG} myoscaffolds and stained with laminin α2 (red). Reduced numbers of SMPCs in laminin dense regions of fibrotic scars (arrow) are observed on *mdx* ^{S} scaffolds. Scale bar, 100μm. **c.** Replicate images from SMPC injected C57-NSG and *mdx*-NSG mice, stained for laminin (green), lamin AC (red), and DAPI (blue). SMPCs in WT and regions of the *mdx* muscle without fibrotic scars (*mdx* ^{NS}) integrated throughout the tissue, while cells were unable to penetrate thickened laminin in fibrotic scars (*mdx* ^{S}). Scale bar, 100μm.

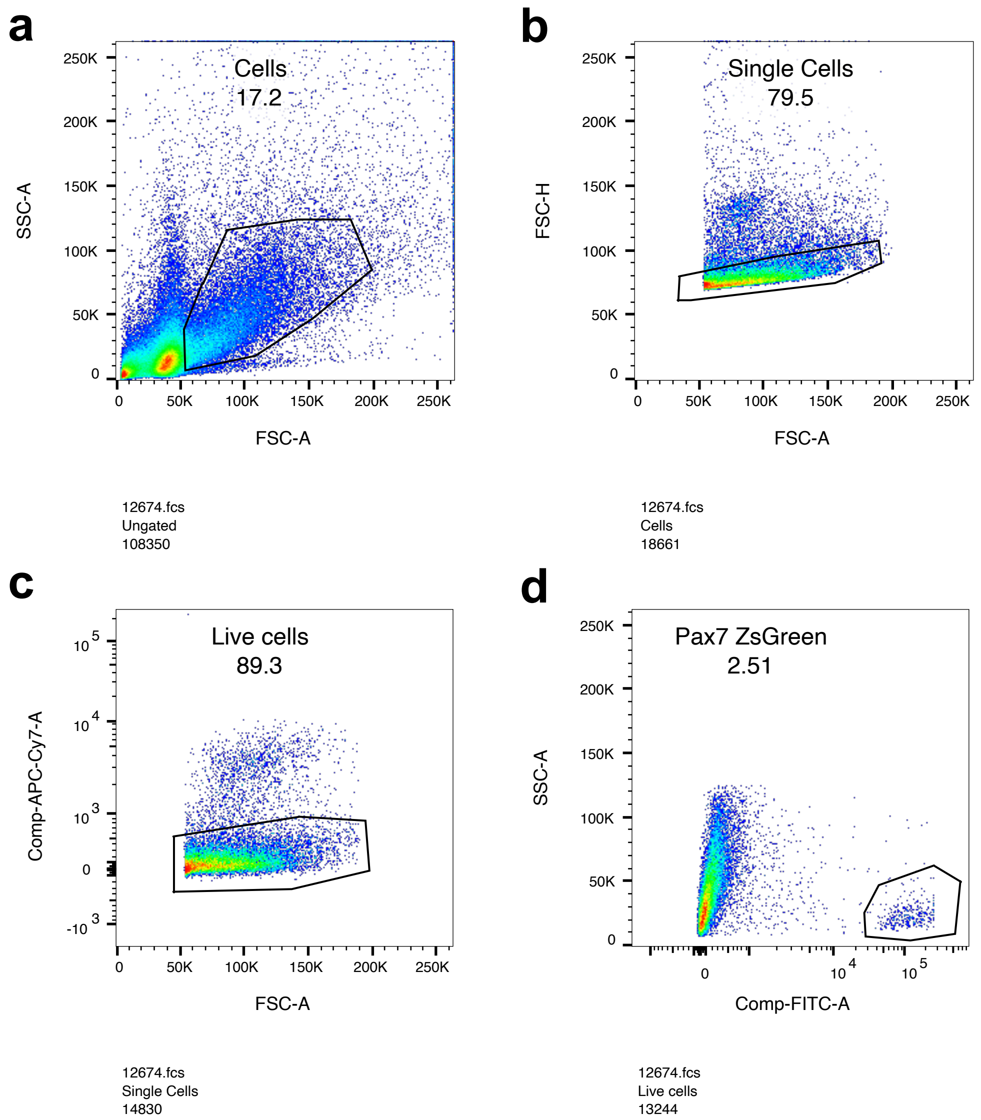

Supplementary Figure 10. FACS-based satellite cell isolation

Representative FACS plots and gating schemes to purify mononucleated satellite cells from Pax7-Cre Rosa26-ZsGreen murine skeletal muscle. Plots show isolation of: **a.** cells (minus muscle debris), **b.** single cells (excluding doublets), **c.** live, lineage negative cells (minus dead cells, erythrocytes, and hematopoietic cells all stained with APC-Cy7), and **d.** pax7+ ZsGreen satellite cells (bright FITC+ gate).

Table 1: List of ECM proteins detected in WT, *mdx*, and *mdx*^{TG} quadriceps muscle samples (n=5/group).

Protein	Gene	Accession Number	Protein Classification
Annexin A1	Anxa1	ANXA1	ECM-affiliated
Annexin A11	Anxa11	ANX11	ECM-affiliated
Annexin A2	Anxa2	ANXA2	ECM-affiliated
Annexin A4	Anxa4	ANXA4	ECM-affiliated
Annexin A5	Anxa5	ANXA5	ECM-affiliated
Annexin A6	Anxa6	ANXA6	ECM-affiliated
Annexin A7	Anxa7	ANXA7	ECM-affiliated
Asporin	Aspn	ASPN	Proteoglycan
Biglycan	Bgn	PGS1	ECM Glycoproteins
Bone marrow proteoglycan	Prg2	PRG2	Proteoglycans
Cartilage intermediate layer protein 1	Cilp	CILP1	ECM Glycoproteins
Cartilage intermediate layer protein 2	Cilp2	CILP2	ECM Glycoproteins
Cathepsin B	Ctsb	CATB	ECM regulator
Cathepsin D	Ctsd	CATD	ECM regulator
Chondroadherin	Chad	CHAD	Proteoglycans
Collagen alpha-1(I) chain	Col1a1	CO1A1	Collagens
Collagen alpha-1(II) chain	Col2a1	CO2A1	Collagens
Collagen alpha-1(III) chain	Col3a1	CO3A1	Collagens
Collagen alpha-1(IV) chain	Col4a1	CO4A1	Collagens
Collagen alpha-1(V) chain	Col5a1	CO5A1	Collagens
Collagen alpha-1(VI) chain	Col6a1	CO6A1	Collagens
Collagen alpha-1(XI) chain	Col11a1	COBA1	Collagens
Collagen alpha-1(XII) chain	Col12a1	COCA1	Collagens
Collagen alpha-1(XIV) chain	Col14a1	COEA1	Collagens
Collagen alpha-1(XV) chain	Col15a1	COFA1	Collagens
Collagen alpha-1(XVI) chain	Col16a1	COGA1	Collagens
Collagen alpha-1(XVII) chain	Col17a1	COHA1	Collagens
Collagen alpha-1(XVIII) chain	Col18a1	COIA1	Collagens
Collagen alpha-2(I) chain	Col1a2	CO1A2	Collagens
Collagen alpha-2(IV) chain	Col4a2	CO4A2	Collagens
Collagen alpha-2(V) chain	Col5a2	CO5A2	Collagens
Collagen alpha-2(VI) chain	Col6a2	CO6A2	Collagens
Collagen alpha-6(VI) chain	Col6a6	CO6A6	Collagens
Fibrillin-1	Fbn1	FBN1	ECM Glycoproteins
Fibromodulin	Fmod	FMOD	Proteoglycan
Fibronectin	Fn1	FINC	ECM Glycoprotein
Galectin-1	Lgals1	LEG1	ECM-affiliated Proteins
Galectin-3	Lgals3	LEG3	ECM-affiliated Proteins
Integrin alpha-7	Itga7	ITA7	ECM regulator
Integrin beta-1	Itgb1	ITB1	ECM regulator
Laminin subunit alpha-2	Lama2	LAMA2	ECM Glycoproteins
Laminin subunit alpha-4	Lama4	LAMA4	
Laminin subunit alpha-5	Lama5	LAMA5	ECM Glycoproteins
Laminin subunit beta-1	Lamb1	LAMB1	ECM Glycoproteins
Laminin subunit beta-2	Lamb2	LAMB2	ECM Glycoproteins

Laminin subunit gamma-1	Lamc1	LAMC1	ECM Glycoproteins
Lumican	Lum	LUM	Proteoglycan
Microfibril-associated glycoprotein 4	Mfap4	MFAP4	ECM Glycoprotein
Nidogen-1	Nid1	NID1	ECM Glycoproteins
Nidogen-2	Nid2	NID2	ECM Glycoproteins
Periostin	Postn	POSTN	ECM Glycoproteins
Prolargin	Prelp	PRELP	Proteoglycan
Protein-glutamine gamma-glutamyltransferas	Tgm2	TGM2	ECM regulator
Proteoglycan 4	Prg4	PRG4	Proteoglycans
von Willebrand factor A domain-containing p	Vwa1	VWA1	ECM Glycoprotein
von Willebrand factor A domain-containing p	Vwa5a	VMA5A	ECM Glycoprotein
von Willebrand factor A domain-containing p	Vwa8	VWA8	ECM Glycoprotein

**Functional Classification**	**Molecular Weight**	*WT 1*	*WT 2*	*WT 3*	*WT 4*	*WT 5*
Other ECM	39 kDa	0	0	0	0	0
Other ECM	54 kDa	16	17	16	18	13
Other ECM	39 kDa	16	15	9	10	9
Other ECM	36 kDa	0	0	0	0	0
Other ECM	36 kDa	6	9	6	7	6
Other ECM	76 kDa	68	79	64	59	48
Other ECM	50 kDa	1	4	0	1	0
Other ECM	43 kDa	28	20	11	12	5
Matricellular	42 kDa	31	42	22	34	19
Other ECM	24 kDa	0	0	0	0	0
Basement Membrane	132 kDa	0	1	0	0	0
Basement Membrane	126 kDa	47	42	35	52	51
ECM regulator	37 kDa	0	0	0	0	0
ECM regulator	45 kDa	2	2	0	2	1
Matricellular	40 kDa	0	0	0	0	0
Fibrillar Collagen	138 kDa	2073	2084	2176	2344	2080
Fibrillar Collagen	142 kDa	8	11	5	15	12
Fibrillar Collagen	139 kDa	494	593	554	569	576
Basement Membrane	161 kDa	33	51	52	52	57
Fibrillar Collagen	184 kDa	54	61	52	53	57
Network Collagen	108 kDa	122	130	123	129	126
Fibrillar Collagen	181 kDa	6	3	4	2	3
FACIT Collagen	340 kDa	91	98	74	107	93
FACIT Collagen	193 kDa	0	0	0	0	0
FACIT Collagen	140 kDa	11	10	13	10	11
FACIT Collagen	156 kDa	1	2	3	3	1
Matricellular	148 kDa	1	1	3	5	6
Multiplexin Collagen	182 kDa	0	0	0	0	0
Fibrillar Collagen	130 kDa	1364	1333	1423	1539	1411
Basement Membrane	167 kDa	60	89	89	108	97
Fibrillar Collagen	145 kDa	79	84	68	77	75
Network Collagen	110 kDa	93	89	86	96	87
Network Collagen	246 kDa	13	17	19	12	27
Structural ECM	312 kDa	40	47	30	41	38
Structural ECM	43 kDa	53	41	35	46	30
Structural ECM	273 kDa	4	3	2	0	1
Matricellular	15 kDa	6	18	12	8	11
Matricellular	28 kDa	0	0	0	0	0
ECM regulator	129 kDa	0	0	0	0	0
ECM regulator	88 kDa	0	0	0	0	0
Basement Membrane	344 kDa	133	117	122	141	125
Basement Membrane	202 kDa	11	9	13	10	4
Basement Membrane	404 kDa	0	3	0	7	7
Basement Membrane	197 kDa	37	43	41	38	36
Basement Membrane	197 kDa	52	66	68	58	60

Basement Membrane	177 kDa	53	58	73	69	72
Matricellular	38 kDa	35	35	24	32	25
Structural ECM	29 kDa	0	4	0	0	0
Basement Membrane	137 kDa	63	73	64	57	45
Basement Membrane	154 kDa	3	4	4	5	4
Matricellular	93 kDa	8	4	0	1	1
Matricellular	43 kDa	45	37	34	35	23
ECM regulator	77 kDa	8	1	0	8	3
Basement Membrane	116 kDa	0	0	0	0	0
Matricellular	45 kDa	4	6	4	3	0
Matricellular	87 kDa	0	0	0	0	0
Matricellular	213 kDa	0	0	0	0	0

mdx 1	*mdx 2*	*mdx 3*	*mdx 4*	*mdx 5*	*mdxTG 1*	*mdxTG 2*	*mdxTG 3*	*mdxTG 4*
8	5	12	11	5	8	6	8	15
30	31	32	30	31	23	28	22	24
20	26	26	23	27	37	28	32	31
3	1	5	2	2	5	7	9	9
16	16	22	19	16	18	12	19	20
69	82	75	70	71	68	49	49	52
16	16	17	14	19	18	16	13	8
34	48	53	37	48	23	21	34	27
41	52	68	62	56	37	40	45	39
2	0	4	0	4	5	4	3	8
9	11	10	11	13	13	12	13	6
40	35	46	40	48	33	33	55	29
0	0	4	3	2	6	3	5	12
3	3	10	3	1	13	9	12	18
0	0	0	0	0	0	8	6	6
1751	1734	2130	1852	1937	2178	2211	2817	2402
8	1	3	8	6	22	127	101	89
697	784	673	745	727	952	918	909	907
51	52	47	55	45	60	66	60	77
74	76	85	67	81	76	70	73	73
161	142	169	130	143	166	152	154	136
3	2	7	3	3	9	11	11	9
97	93	168	93	112	103	117	186	109
4	12	6	2	4	2	2	0	0
17	20	16	16	15	16	20	16	17
4	2	3	5	6	4	4	5	2
4	3	5	5	3	1	1	2	1
0	3	3	2	2	4	4	5	4
1137	1186	1482	1236	1397	1392	1438	1885	1537
89	112	85	101	105	102	103	106	114
90	97	96	94	101	119	115	132	118
115	107	122	110	115	113	94	113	100
23	19	21	8	19	25	14	11	17
36	40	21	35	33	30	31	31	29
27	28	56	29	50	31	32	52	31
39	41	50	61	51	37	16	77	54
22	26	22	19	23	32	31	29	28
3	3	5	6	5	0	0	0	5
2	2	0	0	1	6	2	4	3
0	0	4	3	1	3	2	3	4
177	159	167	166	163	120	125	127	118
26	34	35	32	38	25	17	26	29
5	3	5	5	0	5	3	7	14
54	53	53	59	53	33	37	41	49
93	84	95	75	90	70	58	52	83

86	86	81	92	88	82	70	81	78
58	80	64	53	51	49	39	44	31
0	0	0	0	0	3	2	4	3
69	79	83	70	80	57	47	68	67
18	23	20	15	16	14	7	14	23
34	46	48	42	41	21	20	24	29
34	43	52	30	43	36	28	40	26
14	23	18	19	18	18	28	23	26
0	0	4	2	1	0	0	0	1
6	6	5	5	3	4	2	4	5
0	0	0	0	0	8	4	6	7
0	0	0	1	0	2	0	2	5

	AVERAGES					
mdxTG 5	*WT*	*mdx*	*mdxTG*	**Visualization**	**Fold Change *mdx*/WT**	**Fold Change *mdx*TG/WT**
14	0	8	10			
26	16	31	25		1.925	1.538
31	12	24	32		2.068	2.695
8	0	3	8			
16	7	18	17		2.618	2.500
52	64	73	54		1.154	0.849
9	1	16	13		13.667	10.667
28	15	44	27		2.895	1.750
38	30	56	40		1.885	1.345
0	0	2	4			
10	0	11	11		54.000	54.000
32	45	42	36		0.921	0.802
5	0	2	6			
8	1	4	12		2.857	8.571
2	0	0	4			
2191	2151	1881	2360		0.874	1.097
125	10	5	93		0.510	9.098
843	557	725	906		1.302	1.626
58	49	50	64		1.020	1.310
76	55	77	74		1.383	1.329
141	126	149	150		1.183	1.189
11	4	4	10		1.000	2.833
115	93	113	126		1.216	1.361
0	0	6	1			
14	11	17	17		1.527	1.509
3	2	4	4		2.000	1.800
0	3	4	1		1.250	0.313
4	0	2	4			
1366	1414	1288	1524		0.911	1.078
91	89	98	103		1.111	1.165
117	77	96	120		1.248	1.569
97	90	114	103		1.262	1.146
13	18	18	16		1.023	0.909
27	39	33	30		0.842	0.755
37	41	38	37		0.927	0.893
22	2	48	41		24.200	20.600
35	11	22	31		2.036	2.818
0	0	4	1			
2	0	1	3			
3	0	2	3			
132	128	166	124		1.304	0.975
18	9	33	23		3.511	2.447
6	3	4	7		1.059	2.059
42	39	54	40		1.395	1.036
70	61	87	67		1.438	1.095

67	65	87	76		1.332	1.163
32	30	61	39		2.026	1.291
2	1	0	3		0.000	3.500
62	60	76	60		1.262	0.997
12	4	18	14		4.600	3.500
16	3	42	22		15.071	7.857
24	35	40	31		1.161	0.885
24	4	18	24		4.600	5.950
0	0	1	0			
5	3	5	4		1.471	1.176
6	0	0	6			
3	0	0	2			

Fold Change *mdx* TG/MDX	p value WT, *mdx*	p value WT, *mdx* TG	p value *mdx*, *mdx* TG
1.244	0.005	0.005	0.415
0.799	0.000	0.000	0.003
1.303	0.000	0.000	0.005
2.923	0.019	0.001	0.001
0.955	0.000	0.001	0.678
0.736	0.135	0.166	0.003
0.780	0.000	0.002	0.143
0.605	0.001	0.045	0.005
0.713	0.003	0.069	0.022
2.000	0.089	0.037	0.246
1.000	0.000	0.001	1.000
0.871	0.386	0.156	0.345
3.444	0.088	0.015	0.043
3.000	0.171	0.003	0.009
		0.040	0.040
1.255	0.018	0.171	0.013
17.846	0.055	0.012	0.010
1.249	0.000	0.000	0.000
1.284	0.832	0.023	0.011
0.961	0.001	0.000	0.401
1.005	0.029	0.009	0.930
2.833	1.000	0.000	0.000
1.119	0.246	0.093	0.539
0.143	0.031	0.178	0.047
0.988	0.001	0.002	0.882
0.900	0.049	0.047	0.660
0.250	0.502	0.097	0.001
2.100	0.022	0.000	0.013
1.183	0.138	0.328	0.079
1.049	0.333	0.150	0.465
1.257	0.001	0.000	0.000
0.909	0.000	0.027	0.067
0.889	0.917	0.671	0.592
0.897	0.181	0.023	0.355
0.963	0.698	0.461	0.855
0.851	0.000	0.024	0.568
1.384	0.003	0.000	0.001
0.227	0.002	0.374	0.024
3.400	0.089	0.010	0.030
1.875	0.120	0.001	0.167
0.748	0.000	0.538	0.000
0.697	0.000	0.002	0.012
1.944	0.917	0.180	0.158
0.743	0.000	0.655	0.004
0.762	0.001	0.378	0.015

0.873	0.003	0.070	0.018
0.637	0.002	0.074	0.009
	0.374	0.067	0.002
0.790	0.024	0.974	0.011
0.761	0.000	0.018	0.186
0.521	0.000	0.000	0.000
0.762	0.315	0.418	0.089
1.293	0.000	0.000	0.041
0.143	0.135	0.374	0.187
0.800	0.202	0.611	0.233
		0.001	0.001
12.000	0.374	0.042	0.052
